# Expressing acid-sensing ion channel 3 in the brain alters acid-evoked currents and impairs fear conditioning

**DOI:** 10.1111/j.1601-183X.2011.00683.x

**Published:** 2011-06

**Authors:** V C Vralsted, M P Price, J Du, M Schnizler, A M Wunsch, A E Ziemann, M J Welsh, J A Wemmie

**Affiliations:** † Interdisciplinary Graduate Program in NeuroscienceIowa City, IA, USA; ‡ Department of Internal MedicineIowa City, IA, USA; § Department of PsychiatryIowa City, IA, USA; ¶ Department of Molecular Physiology and BiophysicsIowa City, IA, USA; ** Department of NeurosurgeryIowa City, IA, USA; †† Howard Hughes Medical Institute, Roy J. and Lucille A. Carver College of Medicine, University of IowaIowa City, IA, USA; ‡‡ Department of Veterans Affairs Medical CenterIowa City, IA, USA

**Keywords:** Acid-sensing ion channels, amygdala, ASIC3, fear conditioning

## Abstract

Previous studies on mice with a disruption of the gene encoding acid-sensing ion channel 1a (ASIC1a) suggest that ASIC1a is required for normal fear behavior. To investigate the effects of altering the subunit composition of brain ASICs on behavior, we developed transgenic mice expressing ASIC3 via the pan-neuronal synapsin I promoter. These mice express ASIC3 in the brain, where the endogenous ASIC3 protein is not detected. We found that in ASIC3 transgenic mice, ASIC3 co-immunoprecipitated with the endogenous ASIC1a protein and distributed in the same subcellular brain fractions as ASIC1a. In addition, ASIC3 significantly increased the rate of desensitization of acid-evoked currents in cultured cortical neurons. Importantly, ASIC3 reduced Pavlovian fear conditioning to both context and auditory cues. These observations suggest that ASIC3 can heteromultimerize with ASIC1a in the brain and alter the biophysical properties of the endogenous channel complex. Moreover, these data suggest that ASIC subunit composition and channel desensitization may be critical determinants for ASIC-dependent behavior.

Acid-sensing ion channels (ASICs) are emerging as important molecules in learning, memory and behavior (Coryell *et al.* 2007, 2009; Wemmie *et al.* 2002, 2003, 2004; Ziemann *et al.* 2009). ASICs are members of the degenerin/epithelial Na^+^ channel family that are activated by extracellular acidosis. Multiple ASIC subunits have been identified including ASIC1a, -1b, -2a, -2b and -3. The ASIC1a crystal structure indicates that three subunits unite to form a channel (Jasti *et al.* 2007). In heterologous cells, individually expressed ASIC subunits generate homomeric channels with distinct properties. When co-expressed, different ASIC subunits combine into heteromeric channels, also with distinct properties (Benson *et al.* 2002; Hesselager *et al.* 2004). Although the critical determinants of subunit multimerization are largely unknown, subunits co-expressed in heterologous cells are more likely to combine into heteromultimeric channels than homomultimers (Hesselager *et al.* 2004). Thus, a variety of ASIC subunit combinations are possible with potentially diverse biological functions.

The brain expresses ASIC1a, -2a and -2b, and possibly other subunits. Of these, we have thus far learned the most about ASIC1a, largely because disrupting ASIC1a eliminated currents in brain neurons evoked by acidosis as low as pH 5.0 (Askwith *et al.* 2003; Wemmie *et al.* 2002). At the subcellular level, ASIC1a and ASIC2a are distributed to the surface of neurons and are abundant at the cell body, dendrites and dendritic spines (Wemmie *et al.*, 2002; Zha *et al.* 2006, 2009). Loss of ASIC1a reduced synaptic plasticity (Cho & Askwith 2008; Wemmie *et al.* 2002), eliminated acid-evoked Ca^2+^ increases in dendritic spines (Zha *et al.* 2006) and disrupted context and auditory cue fear conditioning (Wemmie *et al.* 2003). In contrast, expressing ASIC1a in transgenic mice with the pan-neuronal synapsin I promoter increased context fear conditioning above wild-type levels (Wemmie *et al.* 2004). ASICs contribute to other behaviors including unconditioned fear of predator odors and open spaces (Coryell *et al.* 2007) and fear behaviors evoked by carbon dioxide (CO_2_) inhalation (Ziemann *et al.* 2009). Loss of ASIC1a also reduced depression-related behaviors in the forced swim test and other depression models (Coryell *et al.* 2009). An adeno-associated virus vector targeting ASIC1a expression to the basolateral amygdala (BLA) restored some but not all the behavioral deficits in ASIC1a^−/−^ mice (Coryell *et al.* 2008, 2009; Ziemann *et al.* 2009). These observations reveal significant roles for acid-evoked currents in the brain, but much remains to be learned. For example, little is known about the biological importance of ASIC subunit composition and the associated differences in channel kinetics.

ASIC3 (formerly called DRASIC) is the most pH-sensitive of the ASIC subunits (Waldmann *et al.* 1997). ASIC3 is expressed in peripheral nociceptive neurons (Price *et al.* 2001; R.Y. Walder, L.A. Rasmussen, J.D. Rainier, A.R. Light, J.A. Wemmie & K.A. Sluka, submitted) where it contributes to acid-evoked currents (Benson *et al.* 1999; Price *et al.* 2001; Xie *et al.* 2002) and may be important for pain (Price *et al.* 2001; Walder *et al.* 2009). ASIC3 expression in the brain is less well established than other ASIC subunits and might be species-dependent. In the rat forebrain, ASIC3 mRNA was not detected by Northern blot (Waldmann *et al.* 1997). Others subsequently reported the presence of ASIC3 mRNA and protein by polymerase chain reaction (PCR), Western blot and immunohistochemistry in rat brain (Meng *et al.* 2009). ASIC3 expression in human brain was also detected by PCR (Babinski *et al.* 1999). But in mouse brain, ASIC3 mRNA was absent (Chen *et al.* 2002). Furthermore, in mouse brain neurons, endogenous acid-evoked currents did not exhibit ASIC3-like properties, and instead they resembled currents produced in heterologous cells by ASIC1a/ASIC2a heteromers and ASIC1a homomers (Askwith *et al.* 2003). Moreover, following ASIC1a disruption, no ASIC3-like currents were detected in mouse cortex, hippocampus or amygdala neurons (Askwith *et al.* 2003; Wemmie *et al.* 2002; Xiong *et al.* 2004; Ziemann *et al.* 2009). Thus, mouse brain appears to express little, if any, ASIC3. The absence of ASIC3-mediated currents presented an opportunity to manipulate the endogenous channel complex by adding the absent channel subunit to the mix.

In heterologous cells, ASIC3 readily associates with ASIC1a and ASIC2 (Benson *et al.* 2002; Hesselager *et al.* 2004; Xie *et al.* 2002), and heteromultimeric channels composed of ASIC3 and ASIC1a desensitize significantly faster to low pH than either subunit expressed alone (Benson *et al.* 2002; Hesselager *et al.* 2004). Thus, if ASIC3 protein were abundantly expressed in mouse brain, we predicted that it would multimerize with ASIC1a. We further predicted that ASIC3 in the brain would alter the biophysical properties of the endogenous channel complex. Finally, because disrupting ASIC1a and eliminating acid-evoked currents impaired fear behavior (Coryell *et al.* 2007; Wemmie *et al.* 2003; Ziemann *et al.* 2009), we predicted that altering the endogenous channel complex and its biophysical properties would impact fear-related behavior. If these predictions were correct, it would suggest the importance of endogenous ASIC subunit composition and perhaps reveal behaviorally relevant channel properties. To test these predictions, we generated transgenic mice expressing mouse ASIC3 in brain via the pan-neuronal synapsin I promoter.

## Materials and methods

### Mice

The mouse ASIC3 (*ACCN3*) cDNA with two hemagglutinin (HA) epitope-encoding sequences inserted after the first ATG (2HA-mASIC3) was subcloned as a *Not* I restriction enzyme fragment into the expression vector pSTEC-2 (Stec *et al.* 2001; Wemmie *et al.* 2004). In this construct, ASIC3 encoding cDNA was positioned downstream of the synapsin I promoter and a *β*-globin intron, which drives expression in all neurons (Hoesche *et al.* 1993). The transgene was excised from the rest of the prokaryotic vector and microinjected into one-cell fertilized mouse embryos obtained from superovulated C57BL/6J × SJL/J (B6SJL F2) mice using standard procedures (Sigmund 1993; Wemmie *et al.* 2004). Identification of transgene-positive mice was confirmed by PCR using the following primer pair: 5′-GAAGTTGGTCGTGAGGCACT-3′ and 5′-CCTGAGGGAGGTTTAGCGTA-3′ which span the HA sequence and the beginning of the ACCN3 sequence to produce a 314-bp PCR product. For behavioral assays, we used naive 2HA-mASIC3 transgenic mice (F_9_ backcross generations −99.8% C57BL/6J) and wild-type littermates, gender- and age-matched (12–16 weeks old). The sample sizes are reported in the figure legends. The number of males and females was equal and counterbalanced between groups. All animal protocols were approved by the local Institutional Animal Care and Use Committee.

### Protein biochemistry

Brain tissue was homogenized in cold 1% Triton X-100 lysis buffer containing 50 mm Tris pH 7.4, 150 mm NaCl and protease inhibitors [Roche Complete, Mini, ethylenediaminetetraacetic acid (EDTA)-free] using a Potter–Elvehjem homogenizer (either whole brain or dissected brain sections). Protein concentration was determined using the BCA assay (Pierce Protein Research Products, Rockford, IL, USA), 100 µg was run on 7.5% polyacrylamide (Bio-Rad Criterion Tris–HCl precast gel) and transferred to polyvinylidene fluoride (PVDF) membrane for Western blotting. For immunoprecipitation, 500 µg protein lysate was incubated with 30 µl Protein A-Sepharose (Sigma-Aldrich, St. Louis, MO, USA) slurry, 200 µl additional lysis buffer and respective antibody [0.3 µg Santa Cruz F-7 *α*-HA sc-7392, 0.5 µl MTY rabbit anti-ASIC1 serum, 0.5 µg mouse immunoglobulin G (IgG)] for 2 h at 4°C with end-over-end rotation. The precipitate was washed three times with Tris-buffered saline containing 0.5% Tween (TBS-T) and extracted with sodium dodecyl sulfate sample buffer containing dithiothreitol (DTT). Membranes were blocked for 1 h at room temperature with 5% bovine serum albumin (BSA) in TBS-T, then incubated for 2 h at room temperature with primary antibodies diluted in 1% BSA/TBS-T, washed three times with TBS-T, then incubated for 1 h with secondary antibodies. Primary antibodies used were as follows: rabbit polyclonal anti-ASIC1a MTY (C-terminal directed) diluted to 1:5000; mouse anti-PSD-95 diluted to 1:250; rabbit polyclonal anti-GluR2/3 (Upstate) diluted to 1:1000; mouse monoclonal anti-HA (Santa Cruz clone F-7) diluted to 1:1000. Secondary antibodies used were goat anti-mouse or anti-rabbit conjugated to horseradish peroxidase diluted to 1:10 000. Blots were treated with Pierce SuperSignal Western Pico chemiluminescence reagent for visualization. For synaptosome isolation, brain tissue was homogenized in cold synaptosome isolation medium (SIM; 320 mm sucrose, 1.2 mm MgSO_4_, 10 mm HEPES, pH 7.4) containing protease inhibitors. Membranes were isolated by clearing large particles from the homogenate (10-min centrifugation at 1000 *g*), re-extracting the pellet with SIM and then centrifuging the pooled supernatants (40 000 *g* for 30 min). Membranes were resuspended with SIM and layered onto a sucrose gradient (0.85, 1.0 and 1.2 m sucrose in SIM). Gradients were centrifuged at 80 000 *g* for 2 h. The band containing synaptosomes was collected from the 1.0/1.2 interface of the gradient and washed with SIM.

### Electrophysiology

Murine cortical neurons were obtained from individual 1- to 2-day-old pups as described (Wemmie *et al.* 2002). Whole-cell voltage-clamp recordings were made as described previously (Askwith *et al.* 2003; Wemmie *et al.* 2002) at room temperature (20–23°C) using 3–5 MΩ glass pipettes (100 µL, Drummond Scientific, Brommall, PA, USA) prepared with a Flaming/Brown micropipette puller (model P-97/VF, Sutter Instrument, Novato, CA, USA) and recorded with an Axopatch 200B amplifier, digidata 1320A interface Clampex 8.2 software (Axon Instruments, Union City, CA, USA) with a sampling interval of 200 µs and 2 kHz filter. Extracellular pH changes were a rapid solution exchanger (Rapid Solution Changer RSC-200; Biologic, Grenoble, France). Membrane voltage was maintained at −70 mV. Cells were superfused in bath solutions containing (in mm) 100 NaCl, 5.4 KCl, 2 CaCl_2_, 1 MgCl_2_, 10 HEPES, 10 MES and pH was adjusted with TMA·OH. The pipette solution contained (in mm) 10 NaCl, 70 K-gluconate, 10 KCl, 1 MgCl_2_, 10 ethyleneglycoltetraacetic acid (EGTA), 25 HEPES and 3 Na_2_ATP, adjusted to pH 7.3 with KOH. Whole-cell voltage-clamp recordings of acid-evoked currents were obtained from pyramidal neurons in the BLA and central amygdala (CeA) in 300-µm acute vibratome-sliced sections from 10-week old mice and under visual guidance (Nikon FN-MN-H, DIC/infrared optics). Slices were continuously superfused in solution containing (in mm) 115 NaCl, 2.5 KCl, 2.0 CaCl_2_, 1.0 MgCl_2_, 1.25 NaH_2_PO_4_, 26.0 NaHCO_3_ and 10 glucose and equilibrated with 95% O_2_ and 5% CO_2_ (pH 7.3–7.4) at 22°C. Patch electrodes (3–5 MΩ) contained (in mm) 135 K-SO_3_CH_3_, 5 NaCl, 1 MgCl_2_, 0.2 EGTA, 10 HEPES, 3 MgATP and 0.3 NaGTP (adjusted to pH 7.2 with KOH). Currents were filtered at 1 kHz and digitized at 5 kHz. Membrane voltage was maintained at −70 mV.

### Context and auditory cue fear conditioning

Mice were placed in a near-infrared fear-conditioning chamber (Med Associates, Inc., St Albuns, VT, USA). Context training consisted of an 8-min protocol with 3 min of exploration, followed by a 1-s shock (0.75 mA) administered at the end of 3–7 min. Context-conditioned freezing was assessed 24-h later in the same chamber and was defined as an absence of movement other than respiration and scored with VideoFreeze software (MedAssociates Inc., St. Albans, VT, USA). The auditory cue fear-conditioning protocol was similar to context fear conditioning, except training was 14 min, beginning with 3 min of exploration followed by five tone-shock pairings (20-s tone co-terminating with a 2-s shock, 0.75 mA) which were initiated on 4, 6, 8, 10 and 12 min. To assess conditioned freezing to the auditory cue, the context was changed: Smooth flooring was placed over the shock grid, a plastic triangular roof was inserted, peppermint scented extract was added to change odor and lighting was dimmed. Freezing in this context was assessed over a 10-min period, during which the tone was presented continuously throughout 4–6 min.

### Footshock sensitivity

Mice received ten 1-s footshocks at an increasing intensity ranging from 0.01 to 0.2 mA. At each shock intensity, the response rate of 10 trials was quantified to produce a response vs. intensity relationship. Also determined was the vocalization threshold (i.e. footshock intensity at which the mice first vocalized).

### Forced swim test, predator odor and CO_2_-evoked freezing

Forced swim test was performed as described (Coryell *et al.* 2009). Predator odor-evoked freezing was quantified during a 5-min exposure to the fox feces odor [2,5-dihydro-2,4,5-trimethylthiazoline (TMT); Phero Tech, Delta, British Columbia, Canada] as described previously (Coryell *et al.* 2007). Freezing was quantified during a 10-min exposure to 10% CO_2_ as described (Ziemann *et al.* 2009).

### Statistical analysis

Values are expressed as mean ± SEM. Student's *t*-test was used to test for significant differences between two groups. For experiments involving more than two groups or time dependence, analysis of variance (anova) was used. Planned contrast testing within the context of the full anova was used to test relationships hypothesized *a priori* between groups. To assess for differences not hypothesized *a priori*, Fisher's least significant difference (LSD) *post hoc* analysis was used. Probit analysis with 95% confidence intervals was used to test for significant differences in flinching response between genotypes to increasing shock intensity. *P* < 0.05 was considered statistically significant.

## Results

### Expressing ASIC3 in transgenic mouse brain

**Figure 1:**
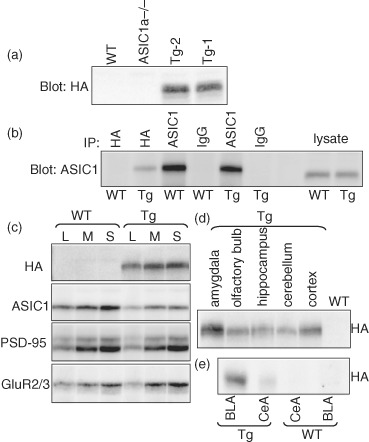
ASIC3 protein expression in transgenic mouse brain (a) Western blotting with the anti-HA antibody detected ASIC3 protein in whole brain extracts from two lines of ASIC3 transgenic mice (Tg-1 and Tg-2), but not in extracts from WT and ASIC1a-null mice. (b) Endogenous ASIC1a co-immunoprecipitated with transgenic ASIC3 protein. Immunoprecipitation with antibody directed against epitopes listed above (or non-specific IgG) and blotted with anti-ASIC1a antibody. At right, Western blot of ASIC1a protein in whole brain lysate. (c) Western blotting of HA-ASIC3, ASIC1a, PSD95 and glutamate receptor (GluR2/3) proteins in brain fractions (L, lysate; M, membranes and S, synaptosome enriched). (d) Transgenic ASIC3 protein in dissected brain regions and (e) in dissected BLA and CeA nuclei.

We generated transgenic mice by using the pan-neuronal synapsin I promoter (Stec *et al.* 2001; Wemmie *et al.* 2004) cloned upstream of the mouse ASIC3 (*ACCN3*) cDNA. We included two HA epitopes engineered into the 5′ coding sequence just after the first ATG, which did not alter ASIC3-mediated current. Two independent lines of ASIC3 transgenic mice were generated (30795/2 and 30795/3). Transgene transmission in these two lines was 52.1 and 46.1%, respectively, suggesting the mutation was not lethal *in utero*. The ASIC3 transgenic mice had normal appearance and were visually indistinguishable from wild-type littermates. Western blotting of whole brain protein lysates confirmed the expression of the ASIC3 protein in the brain of transgenic mice (Fig. 1a). Similar amounts of ASIC3 protein were expressed in the two independent lines. ASIC1 protein was present in the whole brain lysates from ASIC3 transgenics and wild-type littermates (Fig. 1b), suggesting that ASIC3 expression did not disrupt endogenous ASIC1 expression. The ASIC1 antibody is directed against an epitope common to both ASIC1a and ASIC1b; however, in previous studies we were unable to detect ASIC1b protein in brain (Wemmie *et al.* 2002), suggesting the ASIC1 isoform detected here is predominantly ASIC1a.

Because ASIC1a and ASIC3 heteromultimerize in cells where they are co-expressed (Benson *et al.* 2002; Hesselager *et al.* 2004; Xie *et al.* 2002), we hypothesized that the transgenic ASIC3 protein would interact with the endogenous channels containing ASIC1a subunits. Consistent with this hypothesis, we found that endogenous ASIC1a protein co-immunoprecipitated with the HA-tagged transgenic ASIC3 protein (Fig. 1b). Previous biochemical fractionation of brain tissue indicated that ASIC1a is enriched in the cell membrane and synaptosomes (Hruska-Hageman *et al.* 2002; Wemmie *et al.* 2002, 2004). Therefore, we examined whether transgenic ASIC3 exhibited a similar distribution in subcellular brain fractions. We found that like ASIC1a, ASIC3 was enriched in cell membranes and synaptosome-containing brain fractions (Fig. 1c). We also examined transgene expression across different brain regions. We anticipated that the synapsin I promoter would express ASIC3 protein in all neurons and thus in all brain regions. Consistent with this expectation, we found the transgenic protein present in all the dissected brain regions tested (Fig. 1d). However, of the regions tested, the transgenic protein appeared most abundant in the amygdala. ASIC3 protein was present in dissected tissue from both the BLA and CeA nuclei (Fig. 1e). The reason for the amygdala abundance is unclear. The synapsin I promoter may be responsible; we previously obtained similar enrichment of transgenic ASIC1a protein in the mouse amygdala using the same synapsin I promoter (Wemmie *et al.* 2004). Interestingly, endogenous ASIC1a is also abundant in the amygdala (Coryell *et al.* 2007; Wemmie *et al.* 2003, 2004), raising the possibility that endogenous post-transcriptional factors could play a role.

### Effects of ASIC3 on endogenous acid-evoked current

**Figure 2:**
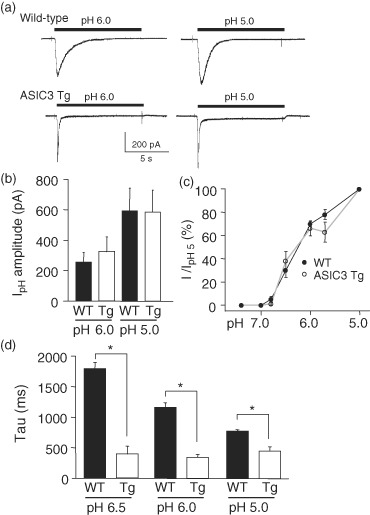
Acid-evoked currents in cultured cortical neurons (a) Representative traces. (b) Peak acid-evoked currents at pH 5 and 6 (*I*_pH_). There was a main effect of pH (*F*_1,46_ = 5.35, *P* = 0.03), but no effect of genotype (*F*_1,46_ = 0.07, *P* = 0.79) or genotype by pH interaction (*F*_1,46_ = 0.106, *P* = 0.75). (c) pH sensitivity of peak current (relative to pH 5.0 −*I/I*_pH 5_ %). There was a significant main effect of pH (*F*_5,118_ = 212, *P* < 0.0001), but no genotype effect (*F*_1,118_ = 1.0, *P* = 0.319) and no genotype by pH interaction (*F*_5,118_ = 1.7, *P* = 0.137). (d) Desensitization rate (*τ*) was significantly increased by ASIC3 expression relative to WTs. Two-way anova revealed a significant genotype by pH interaction (*F*_2,102_ = 23.6, *P* < 0.0001; WT, *n* = 42; Tg, *n* = 66) and planned contrast testing revealed significant differences between genotypes at each pH value (*P < 0.0001).

The ability of ASIC3 to co-immunoprecipate ASIC1a suggested that ASIC3 interacts with endogenous channel subunits. Therefore, we investigated the possibility that ASIC3 would alter acid-evoked currents in neurons from ASIC3 transgenic mice. We found that the amplitude and pH sensitivity of acid-evoked currents in cultured cortical transgenic neurons did not differ from wild-type neurons (Fig. 2a–c). However, the ASIC3 transgenic neurons exhibited a striking increase in the rate of desensitization to low pH (Fig. 2a,d). In addition, unlike wild-type neurons, which desensitize faster with more acidic challenges (Hesselager *et al.* 2004), the desensitization rate of the ASIC3 transgenic neurons did not change with more acidic challenges (Fig. 2d). If ASIC3 expression had produced a separate channel species, we would have expected ASIC3-mediated currents to be superimposed on the endogenous currents. Instead, we saw currents with distinct kinetic properties, suggesting that the transgenic ASIC3 protein heteromulterized with ASIC1a (and possibly ASIC2). Our results closely parallel the effects of co-expressing ASIC3 and ASIC1a in heterologous cells (Benson *et al.* 2002; Hesselager *et al.* 2004); they suggest that transgenic ASIC3 assembled with ASIC1a into heteromeric channel complexes and that these complexes traffic to the cell surface.

**Figure 3:**
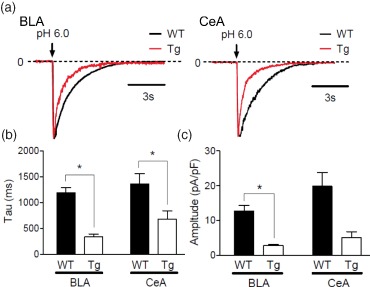
Acid-evoked currents in amygdala neurons in adult brain slices (a) Representative traces. (b) Tau of desensitization. Two-way anova revealed a significant effect of genotype (*F*_1,26_ = 31.2, *P* < 0.001; WT BLA, *n* = 6; Tg BLA, *n* = 9; WT CeA, *n* = 7; Tg CeA, *n* = 8), but no effect of region (*F*_1,26_ = 3.59, *P* = 0.07) and no genotype by region interaction (*F*_1,26_ = 0.37, *P* = 0.55). Planned contrast testing revealed significant differences between genotypes in each region (^*^P < 0.01). (c) Peak current density (pA/pF). Two-way anova revealed a significant effect of genotype (*F*_1,26_ = 7.47, *P* = 0.01; WT BLA, *n* = 6; Tg BLA, *n* = 9; WT CeA, *n* = 7; Tg CeA, *n* = 8) and region (*F*_1,26_ = 6.29, *P* = 0.019), but no genotype by region interaction (*F*_1,26_ = 0.10, *P* = 0.76). ^*^P = 0.04, *post hoc* analysis, LSD.

To test whether amygdala neurons in the adult mice exhibited properties similar to the cultured neonatal neurons, we assessed acid-evoked currents in amygdala slices from 10-week old mice. As in cultured neurons, we found that the rate of desensitization to low pH was increased in transgenic neurons in both the BLA and CeA (Fig. 3a,b). In the brain slices, unlike cultured neurons, peak acid-evoked currents were decreased in the transgenics relative to wild types (WTs) (Fig. 3c), which might be related to the increased desensitization in the transgenics; the *ex vivo* tissue has a reduced ability to maintain a normal pH and hence a fraction of the ASIC channels might be desensitized under basal conditions.

### Behavioral effects of ASIC3 in the brain

**Figure 4:**
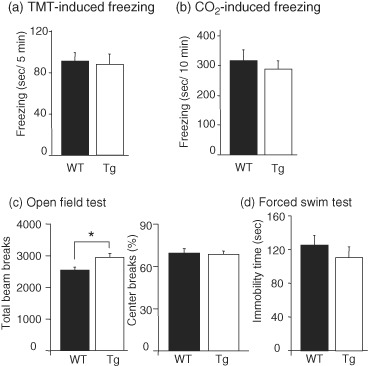
Unconditioned fear and depression-related behaviors in the ASIC3 Tg mice (a) Total TMT-evoked freezing was not affected by ASIC3 expression (*t*_26_ = 0.21, *P* = 0.83; WT, *n* = 14; Tg *n* = 14). (b) Total 10% CO_2_-evoked freezing was unaffected by ASIC3 (*t*_30_ = 0.67, *P* = 0.51; WT, *n* = 16; Tg *n* = 16). (c) In the open-field test, the ASIC3 Tg mice generated more total beam breaks (*t*_20_ = 2.55, *P* = 0.019; WT, *n* = 12; Tg, *n* = 10), but their percentage of center beam breaks did not differ from WTs (*t*_20_ = 0.17, *P* = 0.87). (d) In the forced swim test, ASIC3 did not affect depression-related immobility (*t*_30_ = 0.90, *P* = 0.37; WT, *n* = 16; Tg, *n* = 16).

The effect of ASIC3 on channel desensitization provided an opportunity to assess whether desensitization kinetics might be an important determinant for behavior. Because acid-evoked currents in the ASIC3 transgenic mice desensitized faster, we hypothesized that the shorter time these channels are open would reduce the ability of endogenous ASIC currents to influence behavior. To test this hypothesis, we first examined the effects of expressing ASIC3 in brain on tests of innate fear and anxiety, including predator odor-evoked freezing, CO_2_-evoked freezing and open-field behavior, all of which are impaired by ASIC1a disruption (Coryell *et al.* 2007; Wemmie *et al.* 2003; Ziemann *et al.* 2009). The ASIC3 transgenic mice froze normally to unconditioned stimuli including the predator odor TMT (Fig. 4a) and CO_2_ (Fig. 4b). They also exhibited normal anxiety-related behavior assessed by percentage of center beam breaks in the open-field test (Fig. 4c), despite a slight increase in total beam breaks. Because previous studies demonstrated that ASIC1a disruption produced an antidepressant-like response in the forced swim test (Coryell *et al.* 2009), we also evaluated the ASIC3 transgenic mice in this behavior and found that they did not statistically differ from wild-type controls (Fig. 4d).

**Figure 5:**
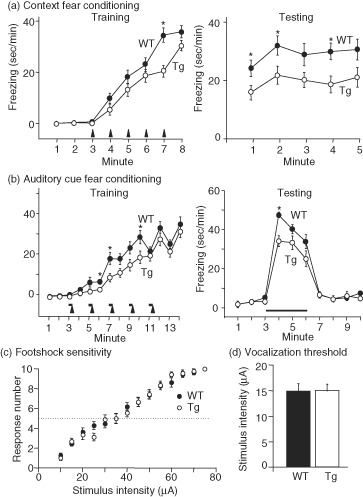
Expressing ASIC3 in brain reduced context and cue fear conditioning (a) Context fear conditioning. During training, there was a significant time by genotype interaction (*F*_7,40_ = 2.31, *P* = 0.045; WT, *n* = 23; Tg, *n* = 25). During memory testing, 24 h later, there was a significant effect of genotype (*F*_1,46_ = 6.88, *P* = 0.012) and time (*F*_4,43_ = 3.83, *P* = 0.01), and no interaction (*F*_4,43_ < 1). (b) Auditory cue fear conditioning. During training, there was a significant effect of time (*F*_13,18_ = 21.5, *P* < 0.001) and genotype (*F*_1,30_ = 4.44, *P* = 0.04), and no interaction (*F*_13,18_ < 1; WT, *n* = 16; Tg, *n* = 16). During memory testing 24 h later, in response to the auditory cue, there was a significant effect of time (*F*_2,29_ = 14.4, *P* < 0.001) and genotype (*F*_1,30_ = 6.40, *P* = 0.017), but no interaction (*F*_2,29_ = 2.1, *P* = 0.14). Repeated measures anova: ^*^indicates significant time-points by estimated marginal means (*P* < 0.05). Arrowheads indicate footshocks. Bars indicate tones. (c) WT and ASIC3 Tg mice demonstrated similar rates of flinching in response to increased shock intensity (10 trials each intensity) [50% response rate (CD_50_) 95% confidence interval WT = 31.0–32.6 µA, Tg = 31.0–32.8 µA; WT, *n* = 11; Tg *n* = 9]. (d) The shock intensity threshold for vocalization also did not differ between genotypes (*t*_18_ = 0.32, *P* = 0.98; WT, *n* = 11; Tg *n* = 9).

Finally, we tested Pavlovian fear conditioning. We chose this paradigm because we know that it is amygdala-dependent (Kim & Jung 2006) and because the ASIC3 transgene was abundantly expressed in the amygdala. Furthermore, our previous data indicate that fear conditioning depends on ASIC1a (Coryell *et al.* 2008; Wemmie *et al.* 2003, 2004). We assessed both contextual fear conditioning and auditory cue fear conditioning. In both cases, ASIC3 expression reduced the acquisition of freezing during training (Fig. 5a,b). In addition, in both paradigms ASIC3 expression reduced conditioned freezing responses during testing relative to wild-type littermate controls (Fig. 5a,b). The behavioral deficit in the ASIC3 transgenic mice was probably not as a result of an inability to feel the footshock, as footshock sensitivity and vocalization threshold were normal (Fig. 5c,d). It was also probably not as a result of an inability to freeze because the ASIC3 transgenics achieved near-normal levels of freezing with additional shocks (Fig. 5a,b). Moreover, their unconditioned freezing response to predator odor and CO_2_ was unaltered (Fig. 4a,b). Together, these findings suggest that ASIC3 expression and the increased desensitization rate of acid-evoked currents reduced fear learning and/or memory.

We tested whether ASIC3 expression altered any of the above-described behaviors in a gender-specific manner. We found no statistically significant gender by genotype interactions. The only behavioral assay where gender produced a significant effect was TMT-evoked freezing; females froze significantly less than males (*F*_1,24_ = 6.27, *P* = 0.02), but there was no effect of genotype (*F*_1,24_ = 0.053, *P* = 0.82) and no genotype by gender interaction (*F*_1,24_ = 0.84, *P* = 0.37). Thus, the effects of ASIC3 on behavior were not gender related.

## Discussion

The lack of ASIC3 in mouse brain provided an opportunity to use transgenically expressed ASIC3 to explore the importance of ASIC subunit composition and the effects of altering channel kinetics on behavior. The transgenic ASIC3 protein associated with ASIC1a in the brain by co-immunoprecipitation. We cannot exclude possible interactions of both proteins in a non-heteromeric state, for example, in large protein complexes. However, the finding that acid-evoked currents in the ASIC3 transgenic neurons desensitized more rapidly indicates that the subunits formed heteromultimers. In previous studies, ASIC3 exerted essentially the same effects on ASIC1a when the two subunits were co-expressed in heterologous cells and in cultured dorsal root ganglia neurons (Benson *et al.* 2002; Hesselager *et al.* 2004; Xie *et al.* 2002). The distribution of ASIC3 closely resembled that of ASIC1a in subcellular brain fractions and did not disrupt ASIC1a expression. Thus, the most striking biochemical/electrophysiological difference in the ASIC3 transgenic mice was the rate at which the acid-evoked currents desensitized to low pH.

Because increased channel desensitization rate would reduce the total charge flow through the channels, we hypothesized that transgenic ASIC3 expression might reduce ASIC1a-dependent behavior. This hypothesis was supported by the results in part; both cue and context fear conditioning were impaired in the ASIC3 transgenic mice. However, ASIC3 expression did not produce all the behavioral deficits seen previously in the ASIC1a^−/−^ mice, where central neurons lack essentially all acid-evoked current (Askwith *et al.* 2003; Wemmie *et al.* 2002; Xiong *et al.* 2004). Behaviors reduced by ASIC1a disruption but not by transgenic ASIC3 expression included unconditioned fear of predator odor and open spaces, CO_2_-evoked freezing behavior and depression-related behavior in the forced swim test. Why ASIC3 expression produced a selective deficit in fear conditioning is not clear. Perhaps the rate of desensitization is especially critical for the ability of the channels to support learning and memory. Alternatively, even though the synapsin I promoter is pan-neuronal, it could be that the ASIC3 transgene does not target all neurons and all circuits equivalently. Because we do not yet know the behaviorally relevant sites of ASIC action at the circuit or neuron level, it is difficult to differentiate between these possibilities. In addition, although we did not see any gross structural abnormalities, we cannot exclude the possibility that subtle developmental changes might underlie the behavioral effects of ASIC3 transgene expression.

These data emphasize the importance of ASIC subunit composition on currents and thereby behavior. By using a genetic method of attenuating charge flow through ASIC channels, we reveal here that fear conditioning is impaired. These results extend our earlier observations that complete ASIC1a disruption impaired and transgenic ASIC1a overexpression enhanced fear conditioning (Wemmie *et al.* 2003, 2004). Thus, we speculate that as a result of genetic variations or pharmacological blockade, variations in ASIC currents might also alter behavior in humans in a dose-dependent manner. An increase in ASIC-mediated current might increase fear memory (Wemmie *et al.* 2004), whereas reducing ASIC current might reduce fear memory. Because fear memories are central to post-traumatic stress disorder (PTSD) (Amstadter *et al.* 2009), these findings suggest ASICs might modify the risk of developing PTSD following a traumatic event.
